# 809 Transportation Access: Impact on Care Service Utilization and Community Integration After Burn Injury

**DOI:** 10.1093/jbcr/iraf019.340

**Published:** 2025-04-01

**Authors:** Edward Santos, Kaitlyn Chacon, Huan Deng, Kara McMullen, Shonali Gaudino, Elizabeth Flores, Caitlin Orton, Karen Kowalske, Chloe Slocum, Mary Slavin, Lewis Kazis, Colleen Ryan, Jeffrey Schneider

**Affiliations:** Spaulding Rehabilitation Hospital, Harvard Medical School; Spaulding Rehabilitation Hospital, Harvard Medical School; Spaulding Rehabilitation Hospital, Harvard Medical School; University of Washington; Spaulding Rehabilitation Hospital, Harvard Medical School; University of Southern California / LA General Burn Unit; University of Washington; University of Texas Southwestern; Spaulding Rehabilitation Hospital, Harvard Medical School; Spaulding Rehabilitation Hospital, Harvard Medical School; Boston University; Shriners Children’s - Boston and Massachusetts General Hospital; University of Washington

## Abstract

**Introduction:**

Transportation access, a social determinant of health, influences health outcomes and community participation. However, transportation is an underexplored factor in burn outcomes. Therefore, this study aims to examine the associations between transportation access, service utilization, and community reintegration after burn injury.

**Methods:**

A multicenter burn longitudinal dataset was analyzed from 2018 to 2024. Self-reported mode of transportation was examined for adults at discharge and 6, 12, 24, and 60 months after injury. The population was categorized into two groups: driving their own vehicle versus other modes of transportation (riding with someone else, public transit, and not applicable). Self-reported utilization of outpatient care services included physical/occupational therapy, peer support, psychological services, and burn-related surgeries. Demographic and clinical variables were compared between groups. Logistic and linear regression analyses, respectively, examined the association between transportation modality and service utilization and community integration (Community Integration Questionnaire – Social Interaction domain) at 6 months.

**Results:**

Of the 563 participants with self-reported transportation data at 6 months, 373 (66.3%) reported driving their own vehicle, 151 (26.8%) riding with someone else, and 31 (5.5%) using public transit. The proportion of those who reported driving their own vehicle increased at each post-injury time point (74.7-83.8%). The driving own-vehicle group was more likely to be White (p=0.002), non-Hispanic/Latino (p=0.001), higher educated, earning a higher income, employed, and married or living with a partner; they also exhibited shorter acute and rehabilitation hospital stays and were less likely to have a range of motion deficit and receive disability income (p< 0.001 for all other comparisons).

Regression analyses revealed no significant associations between driving one’s own vehicle and utilization of the four health care services examined at 6 months. In contrast, regression analysis indicated that driving one’s own vehicle was associated with higher community integration scores (β = 1.23, p< 0.001).

**Conclusions:**

This study found that driving one’s own vehicle was positively associated with community integration but not with utilization of services 6 months after injury. Results suggest that transportation access may play an important role early in community reintegration.

**Applicability of Research to Practice:**

Addressing transportation barriers may contribute to improved community integration outcomes after burn injury.

**Funding for the Study:**

NIDILRR #90DPBU0008 and #90DPGE0004

